# Supplementary material for: Detection of RNA-Dependent RNA Polymerase of Hubei Reo-Like Virus 7 by Next-Generation Sequencing in Aedes aegypti and Culex quinquefasciatus Mosquitoes from Brazil
Source: Viruses. 2019 Feb 10;11(2):147. doi: 10.3390/v11020147 (PMC6410231; doi:10.3390/v11020147)
Supplement: Supplementary file 1 [file viruses-11-00147-s001.pdf]

Table 1. Sequences used in phylogenetic and similarity analyses and their associated details. The abbreviations listed are those used in Figure 2.

Species	Strain name	Country	Host	Abbreviat tion	GenBank access number
Hubei reo-like virus 7	S48	Brazil	*Aedes aegypti*	HRLV 7	MK133923
Hubei reo-like virus 7	mosHB235771	China	Mosquito	HRLV 7	KX884635
Genus Dinovernavirus (9 segments)					
Aedes pseudoscutellaris reovirus	APRV	NA	Mosquito	APRV	YP_443936
Fako virus	CS101	Camaroon	Mosquito	FAKV	YP_009104379
Genus Cypovirus (10 segments)					
Bombyx mori cypovirus 1	Strain I	NA	Moth	BmCPV	AAK20302
Operophtera brumata cypovirus 19	OpbuCPV19	Scotland	Moth	ObRV	ABB17221
Heliothis armigera cypovirus 5	Chinese strain	China	Moth	HaCPV	ABV04399
Genus Oryzavirus (10 segments)					
Raspberry latent virus	RpLV9A	USA	Raspberry	RLV	ADO27688
Rice ragged stunt virus	Thai-isolate	Thailand	Rice	RRSV	AAC36456
Genus Fijivirus (10 segments)					
Fiji disease virus	NA	Australia	Sugarcane	FDV	AAK40249
Mal de Rio Cuarto virus	NA	Argentina	Corn	MRCV	YP_956848
Southern rice black-streaked dwarf virus	HN	China	Rice	SRBV	CBH31251
Genus Coltivirus (12 segments)					
Colorado tick fever virus	Florio	USA	Tick	CTFV	AAG34362
Eyach virus	Fr578	France	Tick	EYAV	NP_620280
Genus Mycoreovirus(10 or 11 segments)					
Cryphonectria parasitica mycoreovirus-1	9B21	USA	Fungi	CpMyRV	AAP45577
Mycoreovirus 3	NA	NA	Fungi	MyRV	YP_392478
Sclerotinia sclerotiorum mycoreovirus 4	SX10	China	Fungi	ScMyRV	ANC52159
Genus Aqueoreovirus(11 segments)					
American grass carp reovirus	AGCRV_PB01155	USA	Fish	AGCV	ABV01040

Fall chinook aquareovirus	GSH1	USA	Fish	FCAV	AQU42726
Genus Orthoreovirus (12 segments)					
Mammalian orthoreovirus 3	MPC/04	China	Mammalian	MOV	ACV52064
Avian orthoreovirus	AVS-B	USA	Avian	AOV	CBX25024
